# The Contribution of Pluripotent Stem Cell (PSC)-Based Models to the Study of Fragile X Syndrome (FXS)

**DOI:** 10.3390/brainsci9020042

**Published:** 2019-02-15

**Authors:** Manar Abu Diab, Rachel Eiges

**Affiliations:** 1 Stem Cell Research Laboratory, Medical Genetics Institute, Shaare Zedek Medical Center, Jerusalem 91031, Israel; manar@szmc.org.il; 2 School of Medicine, Hebrew University of Jerusalem, Jerusalem 9112102, Israel

**Keywords:** fragile X syndrome, unstable repeat diseases, epigenetic gene silencing, DNA methylation, repeat instability, pluripotent stem cells

## Abstract

Fragile X syndrome (FXS) is the most common heritable form of cognitive impairment. It results from a deficiency in the fragile X mental retardation protein (FMRP) due to a CGG repeat expansion in the 5′-UTR of the X-linked *FMR1* gene. When CGGs expand beyond 200 copies, they lead to epigenetic gene silencing of the gene. In addition, the greater the allele size, the more likely it will become unstable and exhibit mosaicism for expansion size between and within tissues in affected individuals. The timing and mechanisms of *FMR1* epigenetic gene silencing and repeat instability are far from being understood given the lack of appropriate cellular and animal models that can fully recapitulate the molecular features characteristic of the disease pathogenesis in humans. This review summarizes the data collected to date from mutant human embryonic stem cells, induced pluripotent stem cells, and hybrid fusions, and discusses their contribution to the investigation of FXS, their key limitations, and future prospects.

## 1. Introduction

Fragile X syndrome (FXS; OMIM#300624) is the most common heritable form of cognitive impairment (1 in 4000 male and 1 in 8000 female births). It is inherited as an X-linked condition and results from a deficiency in the fragile X mental retardation protein (FMRP). Nearly all FXS patients lack FMRP due to a CGG repeat expansion in the 5′-UTR of *FMR1* [1,2,3]. The CGG repeats are located downstream to a CpG island promoter. Rarely, a normal allele in *FMR1* exceeds its standard length to a medium size ((55 < CGGs ≤ 199), premutation or PM) by the addition of CGGs during parent-to-offspring transmission. PM alleles confer a risk of fragile X-associated tremor/ataxia syndrome (FXTAS) and fragile X-associated primary ovarian insufficiency (FXPOI), both of which are thought to result from a combination of toxic gain-of-function RNA and repeat-associated non-ATG (RAN)-translation mechanism [4,5,6,7,8]. The greater the size of PM in the mother, the more likely it will further expand and transform into an FXS-causing mutation (CGGs > 199, full mutation, FM) in the next generation [9]. Once CGGs increase and reach the FM range, they induce aberrant DNA methylation and other changes from active to repressive histone modifications that are typical of densely packed chromatin [1,10,11,12,13,14,15]. This results in *FMR1* transcriptional silencing by abolishing promoter activity. In addition, the greater the allele size, the more likely it will become unstable [2,16,17]. Despite intensive research, the timing and mechanism(s) by which *FMR1* becomes epigenetically modified or unstable are at present far from clear. It is still unknown which repressive histone marks [10,11,12,13,14,15] or chromatin modifying enzymes [18,19,20] are critical for eliciting or maintaining *FMR1* gene silencing. Moreover, it is commonly assumed that gene silencing is facilitated by an RNA-dependent mechanism, although this question remains to be resolved [18,19,20,21]. An additional concern relates to the timing of *FMR1* gene inactivation, which is still a controversial topic [22,23]. Other unresolved issues have to do with the mechanisms underlying repeat instability, and which may differ among germ line, preimplantation stage embryos, and somatic cells. In addition, it is perplexing as to why CGG instability in FXS is at its peak during early fetal development and how this is typically constrained later in life [24,25]. Furthermore, there is conflicting evidence as to the effects of differentiation and methylation in restricting repeat instability in affected tissues [25,26].

Current mouse models, including humanized mice, fail to fully recapitulate the molecular features that are typically associated with the disease in humans. For example, Knock In (KI) mouse models with CGG expansions greater than 199 repeats fail to show hypermethylation of the *FMR1* promoter and inactivate the gene, as observed in humans [27,28,29]. One approach to circumventing this difficulty is to force greater expansions in PM-sized mice by artificially inducing mutations into various DNA processing pathways [30,31,32,33]. These strains have been found useful for investigating the role of DNA repair proteins in promoting CGG instability. However, why these induced expansions do not elicit epigenetic gene silencing in mice remains unclear. An alternative approach for FXS disease modeling is to utilize human pluripotent stem cell (PSC) lines that naturally harbor the disease-causing mutation [21,34,35,36,37,38,39]. This review summarizes the data collected to date on the contributions of currently available PSC model systems to investigate the timing and mechanisms governing epigenetics and repeat instability in FXS, their apparent limitations, and future prospects. The contribution of these cell models to a better understanding of the neural phenotype of the disease, including the effect of RNA/protein toxicity by gain-of-function mechanisms contributed by unmethylated FM alleles, and their therapeutic potential is beyond the scope of this manuscript and can be found elsewhere [40,41,42].

## 2. Currently Available Pluripotent Stem Cell (PSC) Models for Investigating FXS

Pluripotent stem cells are undifferentiated cells that are capable of differentiating into all three embryonic germ layers and their differentiated derivatives [43]. They are transiently present during embryonic development but can also be maintained as established cell lines. PSC lines can be derived from the inner cell mass of blastocysts (embryonic stem cells (ESCs)), primordial germ cells (embryonic germ (EG) cells), or from tumorigenic derivatives of germinal tissues (embryonic carcinoma (EC) cells). The primary advantage of these cell lines is that they can be maintained in vitro indefinitely without undergoing cell senescence while preserving their wide developmental and self-renewal potentials. One fascinating feature of PSCs is their ability to de-differentiate somatic cells by fusion. This is achieved by resetting the epigenetic marks that distinguish somatic cells from undifferentiated embryonic cells and is exhibited by re-establishment of methylation patterns that are characteristic of early embryonic cells [44].

### 2.1. Human Embryonic Stem Cells (hESCs)

**Figure 1:**
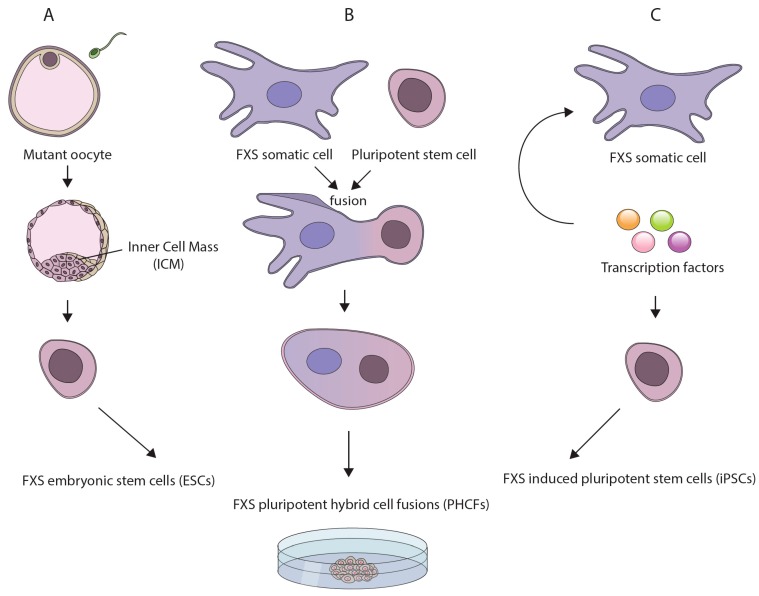
Currently available pluripotent stem cell (PSCs)-based models for investigating the underlying mechanisms for fragile X syndrome (FXS): (**A**) human embryonic stem cell (hESC) lines derived directly from genetically affected FXS embryos following preimplantation genetic diagnosis procedures; (**B**) reprogramming of somatic cells from patients by whole cell/microcell fusion with a pluripotent stem cell, leading to the creation of pluripotent hybrid cell fusions (PHCFs); and (**C**) induced pluripotent cells (iPSCs) derived from patients’ somatic cells by over-expression of a defined set of transcription factors.

Of the various pluripotent cell lines, ESCs best resemble early stage embryos. This is because they are derived from 7-day old spare in vitro fertilized (IVF) human embryos. In addition, they can be established directly from genetically diseased embryos (Figure 1A), which are occasionally available for research purposes from preimplantation genetic diagnosis (PGD) procedures [45,46]. The derivation of human ESCs (hESCs) from FXS affected embryos constitutes a powerful tool for disease modeling since FXS hESCs naturally harbor the disease-causing mutation. This obviates the need to clone lengthy repetitive elements and accurately integrate them into the genome. In addition, FXS hESCs are expected to complement currently available mouse models, which may not accurately replicate the epigenetics or repeat instability aspects of the disease (possibly due to the lack of sequence conservation at the borders of the region that become differentially methylated in patients [47]). Moreover, by more closely resembling preimplantation embryos, they are likely to be a better model for investigating the earliest phases of disease pathology as compared with other human-based model systems such as aborted fetuses, adult postmortem brain samples, or disease unrelated tissues [48,49,50]. Thus far, over a dozen FXS cell lines have been established worldwide and are currently being utilized to address some of the key questions outlined above [42]. However, the accessibility of FXS hESCs is a limiting factor because their derivation is totally dependent on the infrequent availability of PGD derived embryos.

### 2.2. Pluripotent Hybrid Cells Fusions (PHCFs)

An alternative approach to modeling early events in FXS pathogenesis is to de-differentiate patient cells by somatic cell reprogramming. One method to reprogram somatic cells is by fusing them with a PSC [44] (Figure 1B). This can be done by whole-cell fusion or microcell fusion, if the transfer of a single mutant chromosome is desired. The hybrids (hereafter referred to as PHCFs), although chromosomally unbalanced, are considered pluripotent because they preserve their self-renewal and developmental potential as long as they remain undifferentiated. Hence, they constitute a valuable experimental resource for resetting the epigenetic memory of somatic cells/single chromosomes obtained from patients. In the case of FXS this is particularly instructive, since it may allow for reversal of the pathogenic epigenetic modifications that are induced by the CGG expansion in patients’ cells.

### 2.3. Induced Pluripotent Stem Cells (iPSCs)

Another approach for inducing somatic reprogramming of patient cells is to transiently express a small number of transcription factor master regulators [51] (Figure 1C). Quite remarkably, the resulting cell lines, dubbed induced PSCs (iPSCs), closely resemble embryo-derived hESCs in many respects [52,53]. In addition, they are considered superior to PHCFs since they have a normal karyotype and are considered non-tumorigenic. The benefits of iPSC technology are clear. It can be applied, in principle, to any cell type in the body, thus circumventing the obstacle of inaccessibility of spare IVF embryos for hESC line derivation. This technique can be carried out directly on cells obtained from patients, thus enabling any tissue culture laboratory to create pluripotent-based disease models. In addition, the iPSCs derivation procedure typically results in the creation of multiple clones from the same individual (isogenic), that can strengthen the robustness of research findings. Thus far, many iPSCs have been established with the FXS mutation [34,36,37,38,39,54] and are used mostly for the study of the neuronal aspects of the disease [55,56]. One limitation of the iPSC system which is also relevant to PHCFs, is that they are derived by clonal selection. As a result, single clones do not accurately reflect the composition of all alleles within individuals, particularly if the primary cells were derived from subjects who are mosaic for methylation or repeat size [57,58].

## 3. Epigenetics

### 3.1. The Timing of FMR1 Gene Inactivation in FXS

At the time the gene was discovered, it was assumed that CGG expansion is established and transmitted by the mother to the offspring in its methylated and inactive form. However, later studies on chorionic villus samples (CVS) and fetal tissues appeared to suggest that a developmentally regulated process takes place between 8–16 weeks of gestation [22,24,59,60,61]. This led to the view that differentiation-dependent factor(s) are necessary to translate the mutation into gene silencing. Nevertheless, attempts to test this model using patient-derived reprogrammed cells (iPSCs and PHCFs) or FXS hESCs yielded conflicting results. For example, when mouse EC/ES cells were used as recipients to reprogram a fragile X chromosome with a heavily methylated and *FMR1*-inactive gene, this frequently led to de-methylation and restoration of *FMR1* activity in the PHCFs [25]. This not only validated the widespread assumption that *FMR1* epigenetic silencing is triggered by differentiation, but also encouraged researchers to posit that somatic cell reprogramming by iPSC technology could also reverse the epigenetic modifications that are acquired as a consequence of the mutation. In fact, when the first FXS hESC line was established it was found to be completely *FMR1* unmethylated and gene active [35]. However, as more FXS hESC lines were characterized, it became apparent that *FMR1* methylation is not restricted to differentiated cells but can be displayed by undifferentiated cells as well [21,34,62]. Specifically, of the 11 FXS hESCs examined to date, the majority present varying levels of *FMR1* methylation, as high as 65% [34].

Clearly, the wide variability in *FMR1* methylation across different FXS hESCs lines runs counter the claim that gene inactivation is initiated by the end of the first trimester and calls for re-evaluation of the timing of *FMR1* hypermethylation in FXS (reviewed by [23]). In addition, it may suggest that the expansion is not evenly methylated or is not long enough in all cells of the preimplantation embryo. This is consistent with the realization that FMRP loss-of-function may not be the sole mechanism contributing to the clinical phenotype in FXS patients, because mosaicism for allele size and methylation within affected tissues [63] may lead to typical features of FXTAS by toxic gain-of-function mechanisms [57]. An alternative explanation for the extent of variability in *FMR1* methylation among the different FXS hESC lines has to do with the pluripotency state of hESCs, which are heterogeneous in that some cells may reflect a less primitive ground state of pluripotency (primed) as compared to the inner cell mass (ICM) cells in the embryo (naïve) [42,64]. It is thus imperative to directly monitor the expansion size and methylation state of *FMR1* in FXS preimplantation embryos to better understand the dynamics of both expansion size and methylation early in development. In addition, it would be worthwhile establishing FXS hESCs with a FM under naïve conditions to examine whether methylation is abolished under these conditions.

Importantly, when patients’ skin fibroblasts were reprogrammed to generate FXS iPSCs, *FMR1* was consistently hypermethylated and gene inactive [36,37,38]. Moreover, when iPSCs were derived from the skin of an atypical individual who carried an unmethylated FM, reprogramming frequently led to *FMR1* hypermethylation (instead of remaining hypomethylated) and transcriptional silencing in the newly established iPSC clones [39]. On the other hand, in a different study, when iPSCs were derived from blood cells from subjects with an unmethylated FM, *FMR1* remained unmethylated and gene active [54]. The inconsistency in the methylation status of the gene following reprogramming may stem from inter-individual heterogeneity among unmethylated FM carriers due to “clonal selection bias” or may simply result from a difference in the type of cells used for reprogramming (fibroblasts vs. peripheral blood mononuclear cells).

### 3.2. The Role of DNA Methylation in the Silencing Process

When CGGs increase in size and reach the FM range, it results in aberrant DNA methylation in a region that initiates approximately 650-850 nucleotides upstream to the CGGs and extends into intron 1 of the *FMR1* gene [47,65,66]. This disease-associated Differentially Methylated Region, DMR, covers a CpG island (91 CpG sites, GRCh38/hg38 chrX:147,911,574-147,912,682) that overlaps with the *FMR1* promoter and the downstream repeats [47,67]. Upon expansion, the DMR becomes incorrectly methylated presumably by the spread of methylation from the upstream flanking region [47]. This is accompanied by the switch from active (histone acetylations and H3K4me2/3) to repressive (H3K9me2/3, H4K20me3 and H3K27me3) histone modifications [10,11,12,13,14,15], resulting in heterochromatin induction and transcriptional silencing of the *FMR1* gene.

Little is known about the specific role of DNA methylation or the order of events that lead to *FMR1* epigenetic silencing. This is because methylation is only rarely uncoupled from the repressive histone modifications that are associated with silencing in the cell types examined including PSCs [12]. This makes it hard to decipher the role of each of these epigenetic modifications in this process. It is generally assumed that methylation is a relatively late event in the timeline of gene silencing and is responsible for locking up the inactive state. This is because in rare individuals with an unmethylated FM allele, *FMR1* remains transcriptionally active (nonetheless enriched for H3K9me2/3) [14]. Other evidence to support this supposition comes from the treatment of patients’ cells with the demethylation agent 5-aza-dC, which results in partial re-activation of the *FMR1* gene without affecting H3K9me2/3 levels [11,18]. Together, this suggests a mechanism of DNA methylation that is downstream or independent of H3K9me2/3 deposition. With respect to H3K27me3, the results are less consistent. While some FXS PSC lines/clones with a hypermethylated and inactive gene have been significantly enriched for the repressive H3K27me3 mark [68], others were not [69]. The significance of this variation remains to be determined. In addition, it should be mentioned that when *FMR1* was partially re-activated by 5-aza-dC treatment, it led to the recruitment of EZH2, the catalytic subunit of PRC2 (histone methyl transferase that modifies H3K27) and to an increase in H3K27me3 in a manner which is dependent on *FMR1* mRNA expression [18]. The relevance of this to the process of gene silencing, as naturally occurs in patient cells, remains to be determined.

To isolate the functional significance of DNA methylation in FXS, researchers have designed DNA methylation editing tools that exploit the fusion of a catalytically inactive Cas9 with the demethylating enzyme TET1 (dCas9-Tet1) to target methylation at a specific locus in the genome [70]. By designing a single gRNA directed against the CGG repeats, they targeted the dCas9-Tet1 to the *FMR1* locus in multiple FXS patient-derived iPSCs, and efficiently demethylated the repeats [69]. Erasing methylation from the CGGs resulted in hypomethylation of the flanking sequence, increased H3K27 acetylation and H3K4 methylation, and reduced H3K9me2 at the *FMR1* promoter. This unlocked the epigenetic silencing of *FMR1* and restored its activity to nearly normal levels (90%, as compared to 25% after 5-aza-dC treatment [18]) by the recruitment of RNA Pol II to the promoter region. Gene re-activation and demethylation in the FXS iPSC edited clones persisted for at least 2 weeks after inhibition of the dCas9-Tet1 protein in vitro and was sustained in vivo in neurons after transplantation into the mouse brain. This provided the first direct evidence that demethylation of the CGGs is sufficient to re-activate the gene and to switch the region from closed to open chromatin. It would be beneficial if the same programmable dCas9 toolkit approach could be utilized to determine the cause-and-effect relationship between DNA methylation and the other histone marks accompanying the cascade of *FMR1* epigenetic silencing. For example, while there are some hints that H3K9me2/3 is involved in steps preceding DNA hypermethylation, this needs to be experimentally substantiated. By directly tethering the catalytic domain of G9a or Suv39H to the CGGs and depositing H3K9me2/3 in unmethylated FM alleles, it may be possible to uncouple these epigenetic changes to define their causal relationship. Crucially, however, when targeting the CGGs (rather than the flanking sequence) this may lead to off-target effects, given the many CGG repetitive sequences spread throughout the genome [69].

To determine whether CGG expansion is needed at all times to preserve aberrant methylation once silencing is achieved, researchers have taken advantage of XY FXS iPSCs with a heavily methylated expansion to eliminate (as opposed to shorten) the CGGs from *FMR1* with the CRISPR/Cas9 system using gRNAs directed against the flanking sequences [71,72]. By monitoring for changes in *FMR1*, Park et al. demonstrated that excision of the CGGs with a single gRNA completely eliminated aberrant methylation from at least a portion of the *FMR1* promoter (22 CpG sites), switched from H3K9me2 to H3K4me3 enrichments, rescued *FMR1* transcription, and restored protein levels [71]. In a different study Xie et al. were able to reactivate the *FMR1* gene in some of the targeted cells (67% of somatic cell hybrids and only 20% of iPSCs) and increase the mRNA to at least 50% of WT mRNA levels, by efficiently deleting the CGGs using a pair of gRNAs targeting either side of the repeats [72]. However, in the latter study repeat deletion was mostly inefficient or only partly reduced methylation levels to wild type control levels at the promoter (altogether 8 CpG sites). It is hard to reconcile the difference in the methylation changes between the clones. Considering that the precision of the deletion with a single gRNA by Park et al. was not as precise as in the latter report and often led to indels, interpretation calls for caution. Nevertheless, together these studies provide evidence that the repressive marks elicited by the mutation under certain conditions are reversible and need to be established persistently at each DNA replication cycle. Clearly, this should be taken into account when considering the mutation as a potential therapeutic target in non-dividing cells such as affected neurons.

Finally, in a recent study, Haenfler et al. achieved re-activation of a completely methylated *FMR1* gene in an hESC line with approximately 800 repeats by targeting a catalytically inactive Cas9 and transcription activator VAP192 fusion protein directly to the repeats [62]. Using this approach, they induced transcription from the *FMR1* gene despite high levels of methylation in both the promoter and the repeats. This implies that epigenetic silencing in undifferentiated PSCs can be overcome by sustained accessibility of transcription factors to the locus.

### 3.3. The Significance of DNA Hydroxymethylation at the FMR1 Locus

While the role of DNA methylation in *FMR1* epigenetic silencing has been extensively studied, the contribution of 5-hydroxymethylcytosine (5hmC) to this process is less clear and has not been well characterized in differentiated and undifferentiated iPSC-based models. The epigenetic mark 5hmC is produced by DNA demethylation through oxidation of 5mC by the TET family of deoxygenases [73,74]. It acts as an intermediate in DNA demethylation during the conversion of 5mC into cytosine, and thus is implicated in transcriptional activation of genes. There is evidence to show that TET1 acts in differentiated cells as a maintenance demethylase to prevent aberrant methylation spreading into CpG islands [75,76,77]. Consistent with this idea, it was hypothesized that repeat expansion elicits *FMR1* epigenetic silencing by impeding TET-mediated demethylation at the otherwise hypomethylated CpG Island. With this in mind, Esanov and colleagues analyzed the levels of 5hmC at the *FMR1* promoter in post-mortem brain samples, primary fibroblasts and immortalized lymphocytes from FXS and control subjects as well as in vitro differentiated neural progenitors (NPCs) from FXS iPSC and hESCs (WCMC-37) [50]. In their study, the *FMR1* promoter was found to be exclusively 5hmC in primary neurons of FXS patients, suggesting that NPCs established from patient-derived iPSCs or FXS hESCs may not reflect the complete repertoire of epigenetic modifications that are typically found in mature neurons in brains of patients. The reason for the discrepancy between the different cell types is unknown however it may be because 5hmC cannot be well maintained in highly proliferating cells [78]. Hence, the significance of this epigenetic mark to gene silencing is uncertain as it may simply reflect changes in other processes that lead to DNA demethylation such as the activity of repair proteins [79].

### 3.4. The Effect of Differentiation on the Epigenetic Status of the Gene

To date, no simple model can be put forward based on the data collected from FXS PSC-based systems. In some reports, epigenetic modifications were reversed by reprogramming or, conversely, triggered by differentiation [20,21,25]. In other reports, in vitro differentiation, particularly to neurons, had no effect on the methylation status of the gene [54,68]. On the other hand, there is some evidence to suggest that the threshold for silencing lies around 400 repeats in iPSCs derived from individuals with an unmethylated FM, and that this may be the cause of the failure of FXS undifferentiated cells, in general, to methylate the mutant gene [54]. Taking advantage of a selectable reporter system which can be harnessed to identify spontaneous silencing events in *FMR1*, studies have shown that silencing can be induced by increasing the length of the repeats and reversed by contractions [54]. A different study indicated that when the size of the mutation drops below ~400 repeats, but still remains in the FM range, methylation erodes [68]. It would be useful to extend these studies to explore whether PSCs (embryonic or induced) are distinct from other cell types in that they require a greater number of repeats (>199) for methylation to be elicited. In addition, it would be worthwhile exploring whether differentiation can indirectly lead to an increase in methylation levels by selection against unmethylated FMs, as was previously suggested [23,42,68]. In fact, in vitro differentiated iPSC neurons carrying unmethylated FMs presented in addition to FMRP aggregates increased numbers of ubiquitin-positive inclusion bodies as compared to their PM isogenic controls, thus pointing to toxicity of unmethylated FM alleles [54].

### 3.5. Potential Mechanisms for Epigenetic FMR1 Silencing

Mechanistically, researchers are attempting to address how CGG expansion leads to de novo methylation at the 5′-UTR of *FMR1* using FXS hESCs. FXS hESCs may be particularly useful for this type of study since they carry a FM that is frequently unmethylated [34]. This provides a rare opportunity to uncouple the CGG expansion and the epigenetic marks that are elicited post-fertilization. It is generally accepted that untranslated repeat expansions, including the CGGs in *FMR1*, trigger hypermethylation by a common mechanism that relies on incorrect recruitment of silencing complexes/chromatin modifying enzymes to the otherwise hypomethylated CpG island in which they reside. This may result from binding loss/gain of specific DNA-interacting proteins that normally counteract/promote de novo methylation, respectively.

*CTCF and TADs*: One model to explain how CGG expansion leads to hypermethylation of the 5′-UTR of *FMR1* suggests that the insulator-binding protein CTCF differentially binds to the *FMR1* locus next to the repeats thereby protecting the region from the spread of adjacent heterochromatin [80]. CTCF is a zinc finger protein that functions as a barrier against the influence of neighboring regulatory elements. It also blocks the spread of inactive chromatin (for a review see [81]) and separates the genome into independent functional domains (called topologically associated domains or TADs), ranging from 10kb to a few megabases. Consistent with functional studies involving other loci, it was initially hypothesized that when the CGGs expand and reach a pathological range, this leads to the loss of CTCF binding in the immediate proximity of the repeats, resulting in the spread of heterochromatin and promoter inactivation.

To explore the potential role of CTCF as an insulating factor in *FMR1*, researchers took advantage of FXS hESCs and examined whether methylation was coupled with the binding loss of CTCF next to the CGGs. However, no enrichments for CTCF could be detected by Chromatin immuno-precipitation (ChIP) analysis in wild type or expanded cells [34]. This is consistent with an earlier study where the knock-down of CTCF in cells from healthy individuals as well as subjects with an unmethylated FM did not induce *FMR1* hypermethylation [66]. Nonetheless, in a recent genome-wide study using Hi-C datasets from wild type hESCs as well as patient tissues and cell lines, Sun et al. bioinformatically identified chromatin folding patterns that are typical of unstable repeat-associated loci, including *FMR1* [82]. Akin to other unstable tandem repeats, the CGGs in *FMR1* co-localize with the boundaries of TADs, which are typically positioned in CpG-island-dense regions and are occupied by CTCF. Upon expansion, when the gene is epigenetically silenced and heavily methylated, the boundaries of the TADs are altered and CTCF occupancy is abolished (100 kb upstream to the repeats).

While the mechanistic relationship between CTCF binding, TAD boundaries, and epigenetic gene silencing remains to be discovered, it is tempting to suggest that the repeat expansion disrupts the TAD structure by interfering with CTCF occupancy by long range interactions. This could ultimately result in hypermethylation and gene silencing in affected cells, possibly by a shift in the location of the gene from a downstream TAD containing active enhancers to an upstream TAD that is poor in active enhancers. Understanding the cause and effect between repeat expansion, boundary disruption and epigenetic silencing is expected to pave the way for research and provide new insights into the mechanisms that may apply to noncoding repeat expansion pathologies as a whole. On the other hand, hypermethylation may be upstream to the changes in TAD boundaries and the loss of CTCF binding. Alternatively, hypermethylation may be unrelated to these events since the majority of disease-causing repeat loci that co-localize with TAD boundaries in the Sun et al. study, were not coupled with aberrant DNA methylation or gene silencing in patients (21 out of 23 examined loci including *HTT*, *ATN1*, *ATXN3*, *ATXN7*, *CSTB*, *JPH3*, *ZIC2* and *CACNA1A*).

*RNA-mediated epigenetic silencing*: Other models (not necessarily conflicting with insulator-interacting protein binding loss and/or TAD change models), argue for a mechanism that is mediated by RNA [83]. On the basis of accumulating data related to epigenetic gene silencing in other loci, particularly at repetitive elements, it is commonly thought that *FMR1* inactivation is elicited in a way that is RNA-directed. In fact, antisense transcription, siRNAs and R-loop retention have all been put forward as candidates for mediating this process [83]. However, there is no robust experimental evidence to firmly support any of these possibilities. One hypothesized RNA-directed epigenetic silencing mechanism is based on bi-directional transcription, given the identification of two antisense long noncoding RNAs at the 5′-end of *FMR1; FMR4* and *ASFMR1* [80,84]. *FMR4*, is a primate-specific noncoding RNA transcript (2.4 kb) that resides immediately upstream and shares a bidirectional promoter with *FMR1*. Like *FMR1*, it is epigenetically silenced in FXS patients and is up-regulated in PM carriers. However, knockdown of *FMR4* did not affect *FMR1* expression, nor vice versa, suggesting that *FMR4* most likely is not involved with epigenetic regulation of the *FMR1* gene [84]. *ASFMR1*, is an overlapping antisense transcript which initiates from intron 2 of *FMR1* and extends past the CGGs. Similar to *FMR4*, *ASFMR1* is upregulated in PM carriers but is silenced in FM individuals. Given the capacity of long double strand RNA (dsRNA) molecules to be processed into small RNA molecules by the RNAi machinery, it was posited that *ASFMR1* may contribute to epigenetic silencing of *FMR1* by forming dsRNA structures together with *FMR1* mRNA [80]. Another hypothesis is that the propensity of CGG-expanded RNAs to fold into hairpin structures provides a favorable substrate for Dicer activity. Regardless of the mechanism (bi-directional transcription or RNA hairpin structures), Dicer-processed short RNA molecules are thought to attract silencing complexes to the region via pairing directly to the DNA or to nascent RNA transcripts (for instance in pericentromeric regions [85]).

By taking advantage of cell fusion technology to reprogram patient cells with mouse Dicer-depleted ESCs (knocked down with 10% residual mRNA levels), Hecht et al. abolished H3K9me3 marking and hence interrupted the silencing process of *FMR1* directed by cell differentiation [20]. Likewise, by fusing patient cells with a mouse ESC that was completely deficient for the histone methyl transferases Suv39h (double mutants for *Suv39h1* and *Suv39h2*) they successfully abolished heterochromatinization and the transcriptional inactivation of the gene. These findings led to the notion that *FMR1* gene silencing is triggered by the recruitment of Suv39h to the repeats via Dicer-processed CGG-containing small RNAs, resulting in the local induction of heterochromatin (H3K9me3, HP1 and DNA methylation). These authors reported a 2 to 3-fold increase in the number of small RNA molecules with pure CGGs in an FXS hESC with an unmethylated mutation (HEFX), as compared to the wild type control. Their study suggests that *FMR1* transcriptional repression is differentiation-dependent and is mediated by small RNAs working upstream to H3K9me3 heterochromatin induction and DNA hypermethylation. Conversely, in a different study, the knockdown of *Dicer*, *Ago1* and *Ago2* in *FMR1*-expressing FXS hESC lines (WCMC-37 and SI-214) did not prevent epigenetic gene silencing upon differentiation [21], suggesting that gene inactivation may not depend on the RNAi pathway as originally thought. This underscores the need to examine whether over-expression of CGG-pure small RNAs is sufficient to trigger repressive epigenetic modifications in FXS hESCs with an unmethylated FM with/without differentiation.

Other studies exploring the mechanism of *FMR1* gene silencing have pointed to the role of R-loops (three-stranded nucleic structures composed of persistent DNA:RNA hybrids) in the process. For instance, Colak et al., who employed two FXS hESC lines with expansions exceeding 400 CGGs, claimed that hybridization of the mutant mRNA to the promoter and the 5′ flanking region of *FMR1* elicits silencing by inducing a change from active (H3K4me2) to repressive (H3K9me2) histone modifications during neural differentiation [21]. Disrupting the interaction of the mRNA with the CGG-repeat portion of the gene only abolished silencing if induced at a critical time point during differentiation. However, careful examination of the methylation status of *FMR1* in the FXS hESC lines in this study indicated that they were methylated to a certain extent to begin with. Clearly, this should be taken into consideration when addressing the issue of how and when aberrant epigenetic modifications are first set in FXS.

A different RNA-based model relies on the observations that R-loops frequently form across the CGGs between nascent RNA and DNA in *FMR1*-expressing cells with wild type, PM and unmethylated FM alleles [19,86,87,88]. In line with this reasoning, Groh and colleagues suggested that co-transcriptional forming R-loops across the CGGs provide the first trigger for heterochromatinization by directly recruiting the histone methyltransferase G9a and locally depositing H3K9me2 [19]. One assumption is that R-loop stability should be greater in unmethylated FM vs. PM alleles. To confirm this, it would be imperative to stabilize co-transcriptionally formed R-loops in FXS hESCs with an unmethylated FM and monitor for H3K9me2 enrichments. This would attest to a function for R-loops in triggering heterochromatin as proposed in Groh’s study.

However, a study suggests that R-loops may play the opposite role *FMR1* silencing. Ginno and colleagues examined the potential role of R-loops as a counteracting mechanism for de novo methylation at CpG island promoters [89]. Using a computational approach, they showed that promoters embedded within CpG islands are particularly enriched for R-loops. They provided experimental evidence that R-loops in CpG island promoters can prevent de novo DNA methylation by interfering with the recruitment of DNMT3b, the most widely expressed DNMT during early embryo development. This may imply that R-loops normally protect the 5′-UTR of *FMR1* from de novo methylation by preventing the binding of DNMT3b to the region, and that this is hampered upon expansion. It would be of key interest to design studies to interfere with the formation of R-loops across normal or unmethylated FM alleles, to ascribe a function to R-loops in counteracting de novo methylation at the *FMR1* promoter. A summary of potential factors/mechanism(s) that may be involved in *FMR1* epigenetic silencing, as exhibited by the the currently available PSC-based models, is presented in Table 1.

## 4. Repeat Instability

### 4.1. CGG Instability in FXS PSCs

The mechanism governing CGG instability in FXS is far from understood. Nor do we know when and where precisely repeat instability occurs. There is strong evidence to suggest that FM alleles are initially set by expansions of PM alleles pre-zygotically in the female oocyte [90]. However, large expansions and contractions must also arise in the early embryo since FXS patients typically present somatic mosaicism for expansion length even though FM alleles are stable in terminally differentiated patient cells [24,25,63,91]. Consistent with the concept that instability persists post-fertilization is the data from 2-cell stage PM mouse embryos demonstrating both expansions and a high frequency of large contractions [92]. It remains uncertain whether this equally applies to early cleavage human FXS embryos.

The mechanisms underlying CGG instability in the early embryo may be different from those in the oocyte because in the oocyte (a non-dividing cell) repeat instability must be driven by a mechanism(s) that does not depend on DNA replication (repair or recombination). Whereas, in the early cleavage embryo (high proliferating cells) instability may result from both: DNA-replication-dependent and independent mechanisms. CGG instability in the early embryo [92] most likely involves more than one mechanism. This is because expansions and contractions most likely arise from different mutational events [93]. 

Given the tight inverse correlation between CGG hypermethylation and instability in patient cells [94], it was assumed that FXS hESCs, which best resemble embryos before/at the time of implantation, would present extensive CGG instability (large expansions and contractions) when the FM is unmethylated [21,34,35]. Indeed, when the FM allele was hypermethylated and transcriptionally inactive in FXS PSCs, mutation size remained unaltered. On the other hand, when the FM allele was hypomethylated, it manifested a high degree of size variation [25,34,54,68]. Nevertheless, as increasingly more FXS hESC/iPSCs cell lines are being established and monitored for expansion size for longer periods, it has become apparent that unmethylated FM alleles rarely expand in culture [54]. In fact, the only clear example of large expansions was reported in iPSCs at very low frequencies and was dependent on selection [54]. In a different study, when single cell clones were monitored for repeat size and methylation in a given FXS hESC line with >400 repeat expansion (WCMC37), unmethylated FM alleles systematically underwent small increases and decreases in repeat numbers reminiscent of microsatellite instability (gain and loss of a few repeat units) [68]. In no case was there evidence for large step-wise expansions such as the ones described in hESC/iPSC models with other repeat-associated mutations [95,96]. One potential explanation for these small length changes may be due to errant DNA replication through the CGGs, an effect that would be compatible with rapidly dividing cells. In addition, studies have shown that upon extended propagation in an initially heterogeneous cell population, there is selection against unmethylated FM alleles, likely due to toxic gain-of-function mechanisms [54,68]. Altogether, the findings in FXS hESCs and iPSCs with unmethylated FM imply that the currently available FXS PSC lines may not be a suitable model for investigating how FM alleles are initially established. Nevertheless, they may be informative for investigating how DNA replication is impaired by CGG expansion in highly proliferating embryonic cells, and for characterizing the DNA structures (that are formed in vivo) which may predispose the locus to instability.

### 4.2. Potential Mechanisms that May Account for CGG Instability in FXS

*DNA replication and repair*: Over the years, various models have been suggested to explain how the CGGs become increasingly unstable in FXS cells. One DNA replication-based model relates to the slippage of Okazaki fragments during lagging strand synthesis due to unequal distribution of Gs and Cs (positive GC-skew) between the template (C-rich) and the non-template (G-rich) DNA strands in *FMR1*. It is based on that G-rich single stranded DNA tends to form hairpin and other non-canonical intramolecular structures that are more stable and difficult for the cell to resolve than C-rich repeats (CCG) during DNA replication [97,98,99]. According to this model, when the CGG-strand provides the template for lagging DNA synthesis, it induces contractions. This is because the replication machinery skips the secondary structures that are frequently formed by strand-slippage in the template DNA. On the other hand, when the CCG-strand serves as the template for lagging strand synthesis, it favors expansions. This is because the newly synthesized CGGs tend to slip and form hairpin structures in the Okazaki fragments that result in the addition of repeats in newly synthesized DNA [100]. Thus, replication direction and ORI usage relative to the CGGs is likely to dictate whether expansions (ORI located 5′ to the CGGs) or contractions (ORI located 3′ to the CGGs) will be induced. To explain the difference in CGG stability between embryonic (unstable) and adult (stable) tissues, it was postulated that during early embryogenesis there is a switch in ORI usage, leading to a change in the direction of replication across the repeats (origin-shift model).

In line with the replication-based ORI switch model, using a pair of FXS hESCs Gerhardt and colleagues reported a difference in ORI usages between wild type and expanded alleles [100]. By applying an innovative technique for mapping replication origins and measuring fork progression at single molecule resolution (SMARD), they found a switch in the replication direction relative to the repeats combined with fork stalling. Unlike wild type hESCs, they showed that replication initiates mainly from a downstream, rather than an upstream ORI (-53kb), in undifferentiated FXS hESCs, paralleling early embryonic cells. This may indicate that replication across the CGGs can mainly be attributed to lagging strand synthesis and therefore should promote expansions. Gerhardt et al. suggested that once the cells begin to differentiate, replication patterns change and become more similar to those in normal hESCs, which would imply that replication irregularities are developmentally regulated, and by extension that instability is lost with differentiation. In a different study, a polymorphism in the upstream ORI site was identified and associated with the propensity of the CGGs to expand to FMs, thus providing a potential mechanistic link between CGG instability and the failure to activate an upstream ORI by a cis-acting DNA sequence in undifferentiated FXS embryonic cells [101].

It remains unclear whether the change in ORI usages in mutant undifferentiated cells is the cause or the effect of the expansion, and whether this is mechanistically associated with CGG embryonic instability. This is particularly important given the stabilizing effect of de novo methylation, which apparently does not depend on the differentiation state of the cell.

Another suggested mechanism for CGG instability, besides those that rely on a problem in DNA replication, is incorrect DNA repair. It should be noted that as explained above CGG instability may result from both DNA repair and replication problems, depending on the cell type involved. A driving mechanism for error-prone repair, rather than replication, would be particularly pertinent to non-dividing cells like neurons and oocytes. While evidence in human cells is still lacking, accumulating data in PM mice carrying mutations in various DNA repair pathways suggest that CGG instability is promoted by the incorrect recruitment of mismatch repair proteins, particularly MSH2, MSH3 and MSH6, which leads to the activation of error-prone repair pathways [102]. The data related to repeat instability in hESCs/iPSCs in other loci tend to confirm this reasoning. For example, it was shown that when the mismatch repair (MMR) proteins MSH2, MSH3 and MSH6 are downregulated upon differentiation, repeat instability is considerably reduced in hESC/iPSCs with myotonic dystrophy (DM1; CTG repeats), Huntington’s disease (HD; CAG repeats) and Friedrich’s ataxia (FRDA; GAA repeats) mutations [95,96]. In addition, knockdown of *MSH2* and *MSH6* in iPSCs with the FRDA mutation impeded repeat expansions [103]. In fact, genome-wide association studies (GWAS) in humans with these conditions support a relationship between MMR proteins and levels of somatic instability [104,105]. It would be imperative to manipulate the levels of these factors in undifferentiated hESCs/iPSCs with an unmethylated FM to confirm the mechanistic link between these factors and CGG instability in FXS early embryos, as suggested in mice [30,32,102].

*RNA transcription as a mediator of CGG instability*: Regardless of the mechanism (DNA repair or replication), there is a growing consensus that repeat instability is mediated by RNA transcription, potentially through the formation of R-loops. It is assumed that R-loops promote instability by enhancing the formation of unconventional structures such as hairpin and G-quadruplexes by the G-rich unpaired DNA strand in the R-loop. In a recent study, researchers used hESC with wild type and FXS-expanded alleles to finely characterize and precisely map R-loops and single strand DNA displacements across and near the CGG repeats in vivo to better understand the propensity of this locus to become highly unstable [87]. FXS hESCs are particularly useful for this type of study because they often carry unmethylated FM alleles. These authors showed that the CGGs constitute preferential sites (hotspots) for DNA unpairing in normal range alleles. When R-loops are formed, DNA unpairing is more extensive, and is coupled with interruptions of double strand structures by the non-transcribing (G-rich) DNA strand. These interruptions, which were described in an earlier study by Loomis et al. in somatic cells with normal and PM size alleles [86], are likely to reflect unusual structures in the DNA that are hard to process when the CGGs expand significantly. Strikingly, when the *FMR1* gene was hypermethylated and transcriptionally inactive, local unpairing was abolished. This is consistent with the strict inverse correlation between repeat instability and the methylation/transcriptional competence of the gene [87]. It remains unclear whether more complex double strand structures are actually formed in the locus when it is fully expanded and transcribed. Once these structures are identified, they will need to be associated with the induction of DNA damage or replication irregularities to mechanistically link them with CGG instability in FXS.

*Three-dimensional DNA structure*: Another feature that may relate to instability has to do with the 3D structure of the *FMR1* containing region. In the study by Sun et al. (see Section 3.5) it was suggested that repeat instability occurs as a consequence of TADs boundary disruption [82]. Accordingly, short tandem repeats that are located at the TAD boundaries may be inherently unstable. This may be due to their position at the junctions of physical domains, which interferes with mechanisms that normally suppress excessive expansions. Given that TAD boundaries are hotspots for DNA double strand breaks [106], this may predispose the CGGs to error-prone replication or repair. It would be of great interest to explore whether interfering with the insulator function of the TAD boundary, possibly via disruption of a CTCF binding site, would elicit CGG instability (as in the *Atxn7* transgenic mice model [107]), as suggested by this bioinformatic association study.

## 5. Conclusions

Despite intensive research, the timing and mechanism(s) by which *FMR1* becomes epigenetically silenced or extensively unstable in FXS patients remains obscure. This is at least partially due to the lack of appropriate animal and cellular models. Given that mutant PSCs most resemble early embryonic FXS development, they may provide a valuable model system to investigate the dynamic changes that take place in diseased cells at stages that are otherwise inaccessible to research.

Based on the data obtained from these cell lines to date, it is becoming increasingly clear that *FMR1* hypermethylation is not limited to or triggered by differentiation, and that the threshold for silencing in undifferentiated PSCs is most likely higher than assumed and is situated around 400 repeats. Clearly the wide variability in *FMR1* methylation among the different FXS hESC lines calls for re-evaluation of the timing of *FMR1* hypermethylation and suggests that the expansion is not evenly methylated or not sufficiently long enough in all the cells in the preimplantation embryo. In terms of the molecular mechanisms involved in this process, it will be important to identify the earliest epigenetic modification that provides the trigger for heterochromatin induction. The role of RNA transcription and CTCF binding far from the repeats, as well as the significance of the topological organization of the locus, need further study. In addition, given the tight association between CGG instability and the epigenetic status of the repeats, it should become possible to validate the mechanistic association between de novo methylation and CGG instability with the advent of locus-specific epigenetic editing tools. The role of ORI switching and the formation of unusual non-canonical DNA structures in the locus should be further characterized and mechanistically linked to the enhancement of CGG instability in FXS PSCs. In addition, the possibility that the de-methylation of FM alleles can naturally occur in post-mitotic neurons should be explored. Finally, attempting to replicate the results in FXS PSCs and reactivate the silenced gene by modifying the chromatin state of the locus in slow/non- dividing cells will be a major challenge as a therapeutic approach. Even if achieved, the problem of toxic RNA/protein will need to be addressed. Clearly, the currently available FXS PSC models, particularly hESCs and iPSCs, will continue to contribute as complementary powerful systems to fill the gaps in our knowledge of the molecular mechanisms that go awry in FXS at very early stages of development.

## Figures and Tables

**Table 1:** Evidence for the presence (+)/absence (−) of factors that may be involved in *FMR1* epigenetic silencing in various pluripotent stem cell (PSC)-based models of FXS.

	Model System		
	hESCs	iPSCs	PHCFs
**Epigenetic Marks**			
CpG methylation	[21] (+/−) [34] (+/−) [62] (+) [68] (+)	[34] (+) [36] (+) [37] (+) [38] (+) [39] (+) [54] (+) [69] (+) [72] (+)	[20] (−) [25] (−) [26] (+) [72] (+)
5-hydroxymethylation	[50] (−)	[50] (−)	
H3K4me3	[34] (+) [68] (−)	[39] (−) [69] (+)	
H3K9me2/3	[21] (−) [34] (+/−) [39] (+) [69] (+)	[39] (+) [69] (+)	[20] (−)
H3K27me3	[68] (+)	[69] (−)	
**Modifying Enzymes**			
G9a			[20] (+)
Suv39			[20] (+)
DNMT3A			[20] (+)
**Insulator Binding Proteins**			
CTCF	[34] (−) [82] (+)		
**RNA Mediated**			
RNAi pathway	[20] (+)		[20] (+)
R-loops	[21] (+) [87] (+)		
